# Accidents to the Eye

**Published:** 1875-07

**Authors:** 


### ACCIDENTS TO THE EYE.

The number of accidents that are constantly occurring to the eyes of our artizans, throughout the land, is perfectly surprising. In no place like an ophthalmic institution is this fact observable, where only the more serious cases congregate, leaving the untold numbers of slighter injuries to the care of their fellow workmen or the family physician.

The less serious injuries, are those that happen to workmen employed in hammering and chiseling iron, steel, or stone, or to those who work at the emery wheel, where small spiculæ of metal or emery become embedded in the cornea, where they can be readily removed by suitable instruments and a steady hand. The removal of such particles is generally entrusted to some shop mate who attempts the operation with the point of a pen knife or sharp stick, after inflicting more injury to the eye than would have resulted from the continued presence of the foreign substance. The best appliance for the removal of small particles of steel, iron, cinders, stone or emery, consists of a stick, wound upon the end, with dry, fine, white silk ravelings,—the finer the better. This soft pad, brushed backward and forward over the cornea, excites no pain or inflammation, and almost always removes the substance from its hold, by entangling it in the delicate meshes of the silk. Another very good instrument for this purpose, is a paper spud, such as is used by artists in toning down their pencil marks. The end, by being wet in clean water, becomes soft and pliable, and can then be rubbed over the eye and will catch the particle within its folds and so remove it. Sharp pointed instruments should never be used, save by the ophthalmic surgeon, for this purpose, for the reason assigned, that more injury is done the cornea, in scratching its surface, than would have resulted from the mote.

Burns upon the eye lids, from molten metals flying in the face, or from other causes, and cuts about the lids, are exceedingly dangerous, and should always be dressed by a surgeon, else may deformity of the lids ensue, causing great discomfort from inturning lashes or from eversion of the lids.

Lime, from plastering and whitewashing, often drop into the eye, and does great mischief from its caustic effect, burning the inside of the lids and causing them to adhere to the eye-ball by means of fleshy growths that require extensive operations to remove, so as to prevent re-attachments. In such cases, bathe the eye in dilute vinegar, and smear the inside of the lids with olive oil. Should nitric or sulphuric acids come in contact with the eye, as is frequently the case in charging soda fountains or in other chemical exploits, wash the eye, immediately, in a weak solution of bicarbonate of potash,—about four grains to an ounce of water, or with dilute liquor ammonia,—a teaspoonful of liquor ammonia in a small teacup full of water. Where strong alkalies, like caustic potash or soda, come in contact with the eye, it can be neutralized by applications of dilute vinegar and sweet oil dressings, as recommended for the lime. Scalds from hot water or steam, to the eye, can be dressed with olive oil poured directly upon the eye-ball, and dressed externally with pledgets of lint saturated with oil, over which a bandage can be placed.

Blows upon the eye-ball, from ball-throwing play, or from chips of wood flying against the eye, or from falls, are more or less dangerous. If the vision should remain obscured for more than two or three hours after such accidents, and the globe continue painful, while the uninjured eye shuns the light, no time should be lost in consulting an ophthalmic surgeon, for in such cases some lesion has occurred *within* the eye, either dislocation of the lens, rupture of the iris, or perhaps a rupture of some small vessel which discharges its blood into the clear humors of the eye, requiring speedy and proper treatment to prevent the most disastrous consequences to vision.

Incised or penetrating wounds of the eye, from sharp instruments, or from pieces of glass or metal, which puncture the eye but do not remain embedded within its structure, require careful and immediate treatment to prevent the *iris*—the colored membrane in which the pupil is situated—from floating into the wound, and so distorting the pupil. Poultices in such cases are harmful, and patent eye-waters disastrous to an eye so injured, as all such nostrums contain sugar-of-lead, which, when deposited upon an injured cornea, produces a white spot that can never be erased. If an ophthalmic surgeon cannot be reached the same day of the accident, procure one grain of *sulphate of atropia*, and dissolve it in a half ounce of clear rain water, then drop a few drops between the lids three or four times a day, keeping a soft cloth, wet in cold water, bound over the eye, and the patient confined in a dark room until the wound heals. The same advice will hold good with reference to scratches upon the cornea, from briars, the finger nails of a little child, or the scratch of a kitten; all such wounds are very painful, and if not speedily healed result in ulcerations and consequent opacities or "films."

But, by far the most serious in its consequences, is the lodgement of some substance *within* the eye, and which is constantly occurring among all artizans who work in metals and stone, as well as among children who are given to the highly reprehensible habit of exploding percussion caps, which explode with such force as to cause particles of the metal to pierce the eye and find lodgement in its humors or membranes. In such accidents, knowledge as to whether the foreign body is really inside the eye, or whether it has only inflicted its wound and then fallen to the ground, is indispensible. The ophthalmic surgeon, with his ophthalmoscope, alone, can decide this point, for without the aid of such an instrument, nothing whatever can be seen inside the eye-ball. Bullets, and other foreign bodies, have been known to remain in different parts of the body, without doing harm, but not so with the eye. The smallest particle of any substance left therein, is absolutely certain, without the least shadow of a doubt, to destroy vision in the injured one, *and renders the sound eye liable to destruction, through sympathetic inflammation.* Sometimes, after such accidents, the wounded eye remains painfnl for a few weeks, and then gets well, as the patient thinks, so far as pain is concerned; but this quietness is deceptive; it is only a rest previous to another outbreak of pain and inflammation that progresses often to complete destruction of the globe, leaving only a shrunken, deformed stump with the irritating body still inside, to cause periodical attacks of inflammation that is almost certain to ruin the remaining eye. Under these exasperating circumstances, what must be done? *The eye-ball must be removed,* when all cause for sympathetic inflammation is at once ended, leaving the unwounded eye free to regain its former strength and usefulness. If this operation is too long delayed, blindness may ensue in the second eye, notwithstanding the efforts of the ophthalmic surgeon to save it; we cannot thorefore urge too strongly the necessity for prompt and intelligent action in injuries of this character. Extirpation, or removal of the eye-ball is easily and painlessly accomplished, in these days of skillful surgeons and the use of chloroform, and the resulting deformity is completely covered by the insertion of an artificial eye that would never be detected by the casual observer, thus allowing of no excuse for delay in seeking relief.

